# Captain Paul Betowski

**Published:** 1918-09

**Authors:** 


**Captain Paul Betowski** was killed in France on July 2nd. He volunteered to take an automobile full of gas masks to a point on the firing line where there was urgent need. What happened to him is not known but he never returned alive. He had been the assistant surgeon at the Soldiers' Home at Bath for several years. He was born in Waverly 24 years ago.

